# Cervical Adenocarcinoma in the Shadows: The Impact of Missed Screening

**DOI:** 10.7759/cureus.94089

**Published:** 2025-10-08

**Authors:** Ei Mon Mon Kyaw, Baian Alhindawi, Alexandra French

**Affiliations:** 1 Obstetrics and Gynecology, Luton and Dunstable University Hospital, Luton, GBR

**Keywords:** cervical adenocarcinoma, cervical cancer screening, early detection, figo stage iiic2, gynecologic oncology, heavy vaginal bleeding, hpv infection, pap smear, preventive medicine

## Abstract

Cervical adenocarcinoma is a rising concern in women’s health, particularly due to its increasing incidence among younger women and its ability to evade early detection through conventional cytology-based screening. Unlike squamous cell carcinoma, adenocarcinoma often arises higher in the endocervical canal, making it more challenging to detect, especially in patients who default on cervical screening.

We report the case of a 39-year-old British lady who presented with severe anemia secondary to heavy vaginal bleeding. She had never attended cervical screening despite living in a country with an established national screening program. Clinical examination revealed a friable cervical mass. Initial investigations revealed profound microcytic hypochromic anemia requiring urgent transfusion. Imaging with CT demonstrated a bulky, irregular cervix with suspicious pelvic lymphadenopathy, while MRI confirmed a large cervical mass (7.4 × 6.5 × 6.0 cm) with parametrial extension and para-aortic nodal disease, consistent with International Federation of Gynecology and Obstetrics (FIGO) Staging stage IIIC2 cervical carcinoma. Histopathology revealed Human Papillomavirus (HPV)-associated adenocarcinoma, Silva pattern C, with strong carcinoembryonic antigen (CEA) positivity and no lymphovascular invasion. Following multidisciplinary team review, the patient was referred for definitive chemoradiotherapy.

This case illustrates how defaulted cervical screening can result in delayed diagnosis and advanced disease, even within high-resource healthcare systems. It highlights the diagnostic challenges specific to adenocarcinoma, the limitations of Pap smear in detecting glandular lesions, and the importance of HPV-based screening and public education to improve participation. Early recognition of abnormal bleeding and systematic evaluation with MRI and histopathology are crucial for timely diagnosis and management.

## Introduction

Cervical cancer is a significant global health concern, ranking as the fourth most commonly diagnosed cancer and the fourth leading cause of cancer-related deaths among women. In 2020 alone, more than 600,000 new cases and approximately 340,000 deaths were reported, with the vast majority occurring in low- and middle-income countries where access to screening and treatment remains limited [1,2]. This stark disparity underscores the ongoing global inequities in cervical cancer prevention and control.

In contrast, high-income countries such as the United Kingdom have seen substantial reductions in cervical cancer incidence and mortality, largely due to the implementation of national human papillomavirus (HPV) vaccination initiatives and well-organized cervical screening programs [3]. However, challenges persist, including under-screening and barriers to participation, which can delay diagnosis even in well-resourced healthcare systems. The* *factors such as embarrassment, fear, perceived discomfort, and lack of awareness continue to limit screening uptake, even in countries with free and accessible services [4]. These barriers must be actively addressed through culturally sensitive outreach, education, and patient-centered engagement strategies to maximize the effectiveness of cervical cancer prevention programs.

Despite widespread adoption of cervical screening and HPV vaccination globally, significant disparities persist in coverage, follow-up, and outcomes. The challenge lies not only in expanding access but also in improving the accuracy of screening, particularly in detecting glandular lesions and reaching under-screened populations [4]. Emerging strategies such as mailed HPV self-sampling kits have shown promise in increasing screening uptake, especially among under-screened women and underserved communities [5-7]. These self-collection approaches, when paired with targeted communication and support, may help reduce diagnostic delays and improve participation across diverse populations [6].

Current guidelines in the United Kingdom recommend cervical screening with HPV primary testing followed by cytology triage for positive results, and colposcopic evaluation when indicated [3]. This approach is highly effective in detecting squamous cell carcinoma and its precursors. However, it may be less sensitive for glandular lesions that arise higher in the endocervical canal [5]. In our case, the patient had never engaged with cervical screening and presented with symptoms of advanced disease. Her diagnosis was made based on clinical examination, biopsy of the cervical mass, and MRI for staging, rather than through routine screening pathways. This contrast highlights the challenges of missed screening and the importance of alternative strategies for detection, particularly for cervical adenocarcinoma. Pan-specific cervical cancer screening programs typically incorporate a combination of cytology-based screening (Pap smear), high-risk HPV DNA testing, and colposcopic assessment for abnormal results. Recent advancements, including self-sampling kits and dual-stain cytology, are being integrated to improve the detection of adenocarcinoma precursors and reach under-screened populations [1,4-6].

Squamous cell carcinoma is the most common histologic subtype of cervical cancer, but the incidence of adenocarcinoma has been increasing, now comprising 10%-25% of cases [3]. This subtype arises higher in the endocervical canal and is often more difficult to detect through conventional cytology-based screening [8], leading to delayed diagnoses and more advanced disease at presentation [3]. Immunohistochemical biomarkers such as p16, carcinoembryonic antigen (CEA), and p63 can aid in distinguishing between glandular and squamous subtypes, particularly when morphology is ambiguous [9-11].

More recently, the classification of cervical adenocarcinoma has evolved, differentiating between HPV associated and HPV-independent types. HPV-independent adenocarcinomas, such as the gastric type, carry a worse prognosis and tend to show resistance to conventional therapies [12,13].

We present a case of HPV-associated cervical adenocarcinoma in a 39-year-old British woman who presented with severe anemia due to abnormal uterine bleeding. She had never participated in cervical screening, highlighting the risks of under-screening even in high-income countries with organized screening systems. This case underscores the need for improved awareness, outreach, and early detection strategies to reduce morbidity associated with glandular cervical neoplasia.

## Case presentation

A 39-year-old British woman presented to the accident and emergency department with a four-day history of profuse vaginal bleeding, associated with dizziness, fatigue, and reduced exercise tolerance. She appeared pale but was hemodynamically stable on admission. On further questioning, she disclosed a 12-month history of irregular and progressively worsening vaginal bleeding. Initially infrequent, the bleeding episodes had become more prolonged, heavier, and less predictable over time. Notably, she reported recent episodes of postcoital bleeding and intermittent foul-smelling vaginal discharge, which she had previously attributed to a hormonal imbalance and for which she did not seek medical attention.

Her menstrual history was unclear, but she described heavier and more irregular cycles over the past year. She denied pelvic pain, weight loss, or constitutional symptoms. She had two previous spontaneous vaginal deliveries, no history of miscarriage or termination, and no prior gynecological investigations. She was not using any contraception and had never participated in the NHS cervical cancer screening program, despite being eligible. She reported no significant medical or surgical history and was a non-smoker.

On speculum examination, a large, friable, exophytic cervical mass measuring approximately 4 × 4 cm was visualized, bleeding readily on contact. Involvement of the upper vaginal wall was suspected. Bimanual examination suggested a firm, immobile cervix with possible parametrial involvement. A punch biopsy was obtained from the cervical lesion. Red flag signs such as postcoital bleeding, irregular heavy bleeding in a woman over 35, and contact bleeding from a visible cervical mass warranted urgent referral and further investigation, highlighting missed opportunities for earlier detection.

Initial laboratory evaluation revealed profound anemia with a hemoglobin of 44 g/L, microcytic indices, and thrombocytosis (Table 1). Renal, liver, and electrolyte panels were within normal limits. The patient was managed initially with intravenous tranexamic acid (1 g every 8 hours), and hemostasis was achieved. She received a total of four units of packed red blood cells over 48 hours, alongside intravenous fluids and close monitoring. Vital signs were stabilized prior to further diagnostic evaluation.. Follow-up blood counts demonstrated a rise in hemoglobin to 96 g/L, with partial correction of red cell indices and normalization of platelet count (Table 1).

**Table 1:** Serial full blood count (FBC) results from admission and after blood transfusion. This table displays the patient’s hematological parameters upon admission and following blood transfusion on Days 2 and 4 (March 10, 2025, and March 12, 2025, respectively). Initial results demonstrate severe microcytic hypochromic anemia with low hemoglobin, RBC count, and MCV/MCH, consistent with iron deficiency anemia. Progressive improvement in hemoglobin and red cell indices is noted post-transfusion. White blood cell and platelet counts remained within or slightly above reference ranges throughout. MCV, mean cell volume; MCH, mean cell hemoglobin; NRBC, nucleated red blood cells

Parameter	Reference range	On admission	March 10, 2025	March 12, 2025
White blood cell count	4-11 × 10⁹/L	11.1 × 10⁹/L	11.1 × 10⁹/L	12.8 × 10⁹/L
Hemoglobin	120-160 g/L	44 g/L	74 g/L	96 g/L
Platelets	150-450 ×10⁹/L	469 × 10⁹/L	391 × 10⁹/L	341 × 10⁹/L
Mean platelet volume (MPV)	7.8-11 fL	8.2 fL	8.0 fL	7.9 fL
Red blood cells (RBCs)	4-5.2 × 10¹²/L	2.0 × 10¹²/L	3.1 × 10¹²/L	3.7 × 10¹²/L
Hematocrit	0.36-0.46 L/L	0.20 L/L	0.23 L/L	0.29 L/L
Mean cell volume (MCV)	80-100 fL	61 fL	75 fL	80 fL
Mean cell hemoglobin (MCH)	27-32 pg	22 pg	24 pg	26 pg
Mean cell hemoglobin concentration (MCHC)	280-355 g/L	214 g/L	319 g/L	331 g/L
Red cell distribution width (RDW)	11.8%-14.8%	13.1%	23.9%	22.1%
Neutrophils	2-7 × 10⁹/L	7.0 × 10⁹/L	7.81 × 10⁹/L	8.73 × 10⁹/L
Lymphocytes	1-3 × 10⁹/L	1.86 × 10⁹/L	2.15 × 10⁹/L	2.79 × 10⁹/L
Monocytes	0.2-1 × 10⁹/L	1.01 × 10⁹/L	1.01 × 10⁹/L	0.97 × 10⁹/L
Eosinophils	0-0.4 × 10⁹/L	0.00 × 10⁹/L	0.00 × 10⁹/L	0.15 × 10⁹/L
Basophils	0.02-0.1 × 10⁹/L	0.12 × 10⁹/L	0.12 × 10⁹/L	0.15 × 10⁹/L
NRBC	0-0.5 × 10⁹/L	-	<0.5 × 10⁹/L	<0.5 × 10⁹/L

Viral screening for HIV, hepatitis B, and hepatitis C was negative (Table 2). 

**Table 2:** Viral screening results. Screening for HIV, hepatitis B, and hepatitis C was negative.

Test	Method	Result
HIV 1 and 2 antigen/antibody	Roche COBAS 8000	Not detected
Hepatitis B surface antigen	Roche COBAS 8000	Not detected
Hepatitis C antibody	Roche COBAS 8000	Not detected

A computed tomography (CT) of the thorax, abdomen, and pelvis (CT TAP) and an MRI of the pelvis were performed, and the summary of findings is shown in Table 3.

**Table 3:** Summary of radiological findings. CT raised suspicion of a cervical malignancy with nodal involvement, but was limited in tissue characterization. MRI confirmed a bulky cervical carcinoma with parametrial extension and para-aortic nodal metastases, consistent with FIGO stage IIIC2 disease. FIGO, International Federation of Gynecology and Obstetrics

Feature	CT thorax/abdomen/pelvis	MRI pelvis
Cervix	Bulky, irregular cervix; limited tissue characterization	Bulky cervical carcinoma (7.4 × 6.5 × 6 cm) involving the cervix and lower uterine segment
Parametrial involvement	Not well defined	Parametrial infiltration confirmed
Uterine fundus	Linear soft tissue bands of uncertain significance	T2 intermediate signal likely representing an incidental clot
Lymph nodes	Enlarged common iliac nodes (R 8 mm, L 11 mm) and left obturator nodes (up to 8 mm), suspicious for metastasis	Enlarged obturator, common iliac, and para-aortic nodes confirming metastatic nodal disease
Thorax/abdomen/other viscera	Lungs clear, no visceral lesions, no skeletal abnormalities	No invasion of the bladder, rectum, or adnexae
Impression	Enlarged cervix - possible primary carcinoma vs prolapsed fibroid; enlarged pelvic nodes suspicious for metastasis; MRI correlation recommended	Cervical carcinoma with parametrial extension and nodal metastases to the para-aortic region - FIGO stage IIIC2

**Figure 1:**
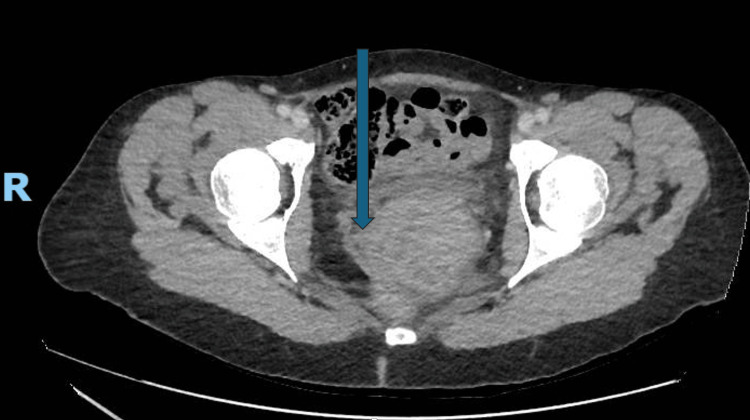
Axial CT pelvis with contrast showing enlarged cervical mass.

Initial imaging with CT TAP revealed a bulky, irregular cervix with soft-tissue bands extending from the uterine fundus. Although initially raising the possibility of a prolapsed fibroid or polyp, the imaging also demonstrated multiple enlarged pelvic lymph nodes-including right and left common iliac and obturator nodes-raising concern for primary cervical carcinoma with nodal involvement. No distant metastases or abnormalities were seen in the lungs, liver, spleen, pancreas, kidneys, or bones (Figure 1). The CT TAP revealed a bulky, irregular cervix with suspicious soft-tissue thickening. However, the exact dimensions of the lesion were not specified in the report. The presence of pelvic lymphadenopathy prompted further evaluation with pelvic MRI for detailed local and nodal staging.

**Figure 2:**
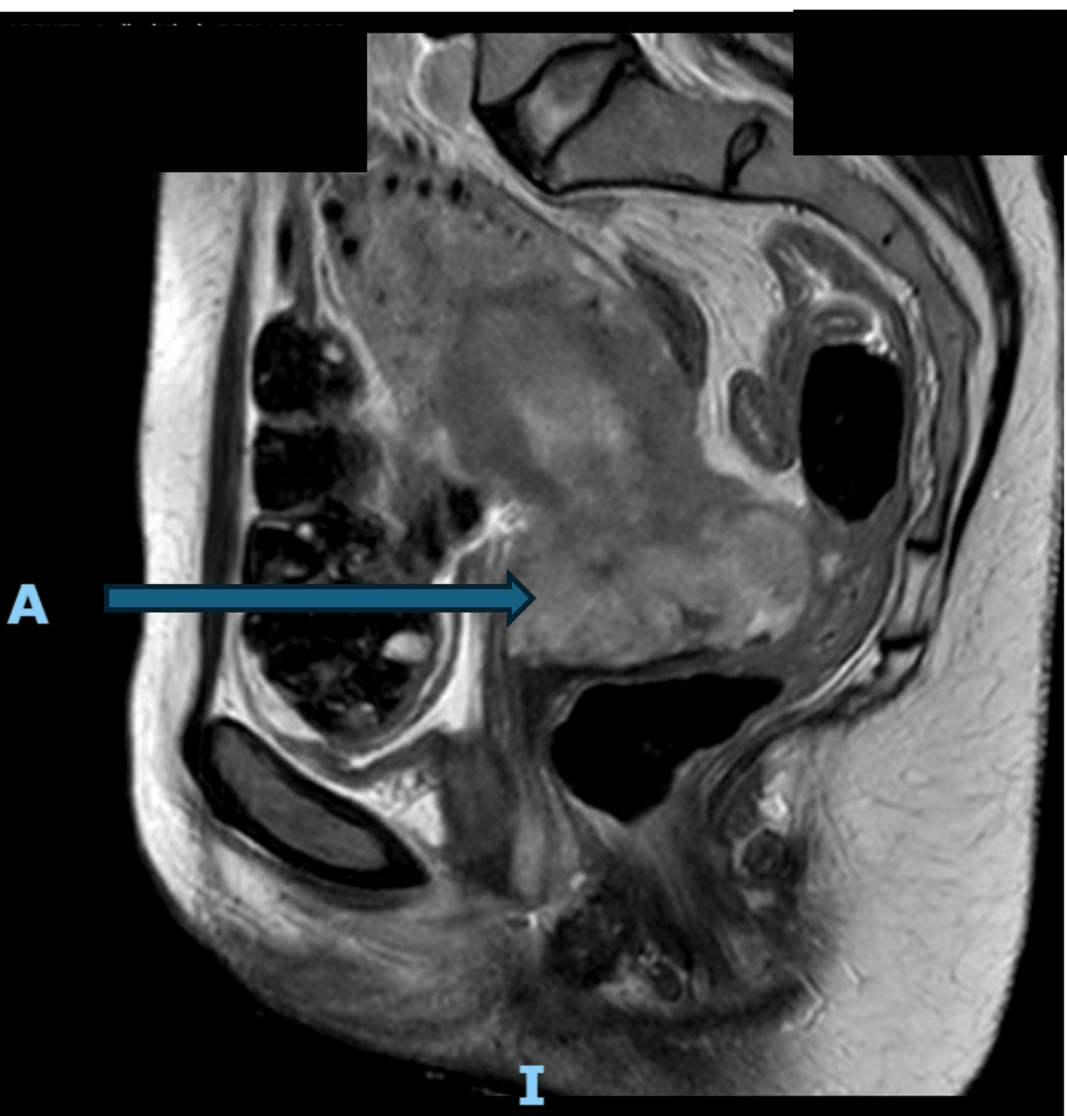
Sagittal T2-weighted pelvic MRI showing a bulky cervical tumor. Sagittal T2-weighted MRI demonstrates a heterogeneously hyperintense mass replacing the cervix (arrow). The lesion distorts the cervical anatomy and extends toward the lower uterine segment, with evidence of parametrial invasion. The rectum and bladder are compressed but not invaded.

**Figure 3:**
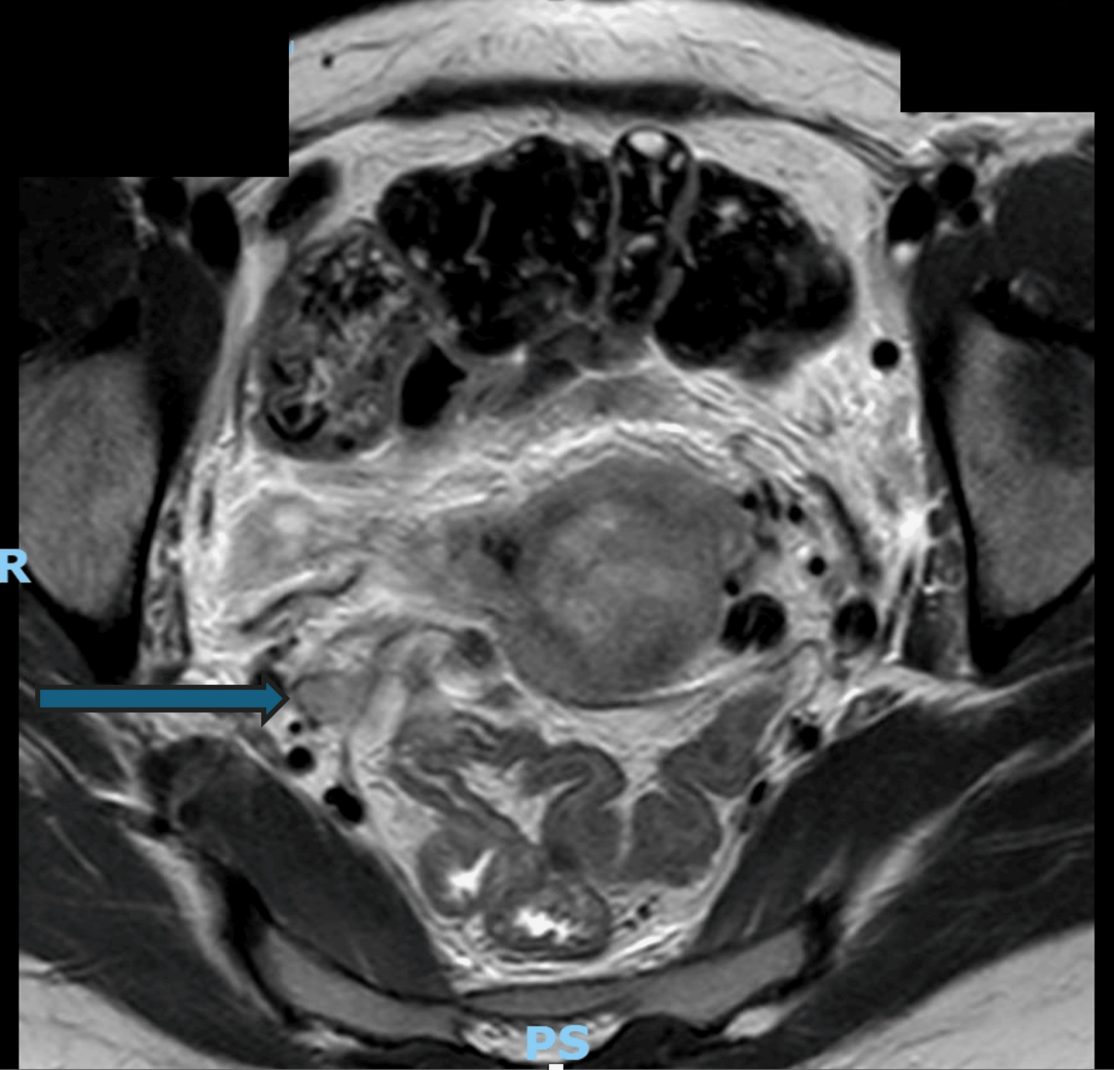
Axial T2-weighted pelvic MRI showing lymph node involvement. Axial T2-weighted MRI demonstrates an irregular, heterogeneously hyperintense cervical mass. The blue arrow indicates enlargement of the right internal iliac lymph node.

Pelvic magnetic resonance imaging (MRI) was performed for more accurate local and nodal staging. It confirmed a large cervical mass measuring 7.4 cm craniocaudal × 6.5 cm anteroposterior × 6.0 cm transverse, with intermediate T2 signal intensity and heterogeneous enhancement, consistent with a malignant tumor. The lesion demonstrated transmural invasion with extension into the parametrial tissues but no evidence of direct invasion of the rectum, sigmoid colon, bladder, or adnexa. Multiple enlarged pelvic and para-aortic lymph nodes were noted, supporting metastatic nodal disease. These findings were consistent with FIGO (International Federation of Gynecology and Obstetrics) stage IIIC2 cervical carcinoma according to FIGO Staging 2021 (Figures 2, 3) [14].

Biopsy from the cervical mass showed fragments of a necrotic tumor composed of sheets of round to polygonal cells with abundant eosinophilic to focally clear cytoplasm, moderately pleomorphic nuclei, and occasional nuclear palisading. Mitotic activity was brisk with atypical mitotic figures, and areas of tumor necrosis and desmoplastic stromal reaction were present. No definite gland formation or papillary architecture was identified, ruling out well-differentiated adenocarcinoma patterns.

**Figure 4:**
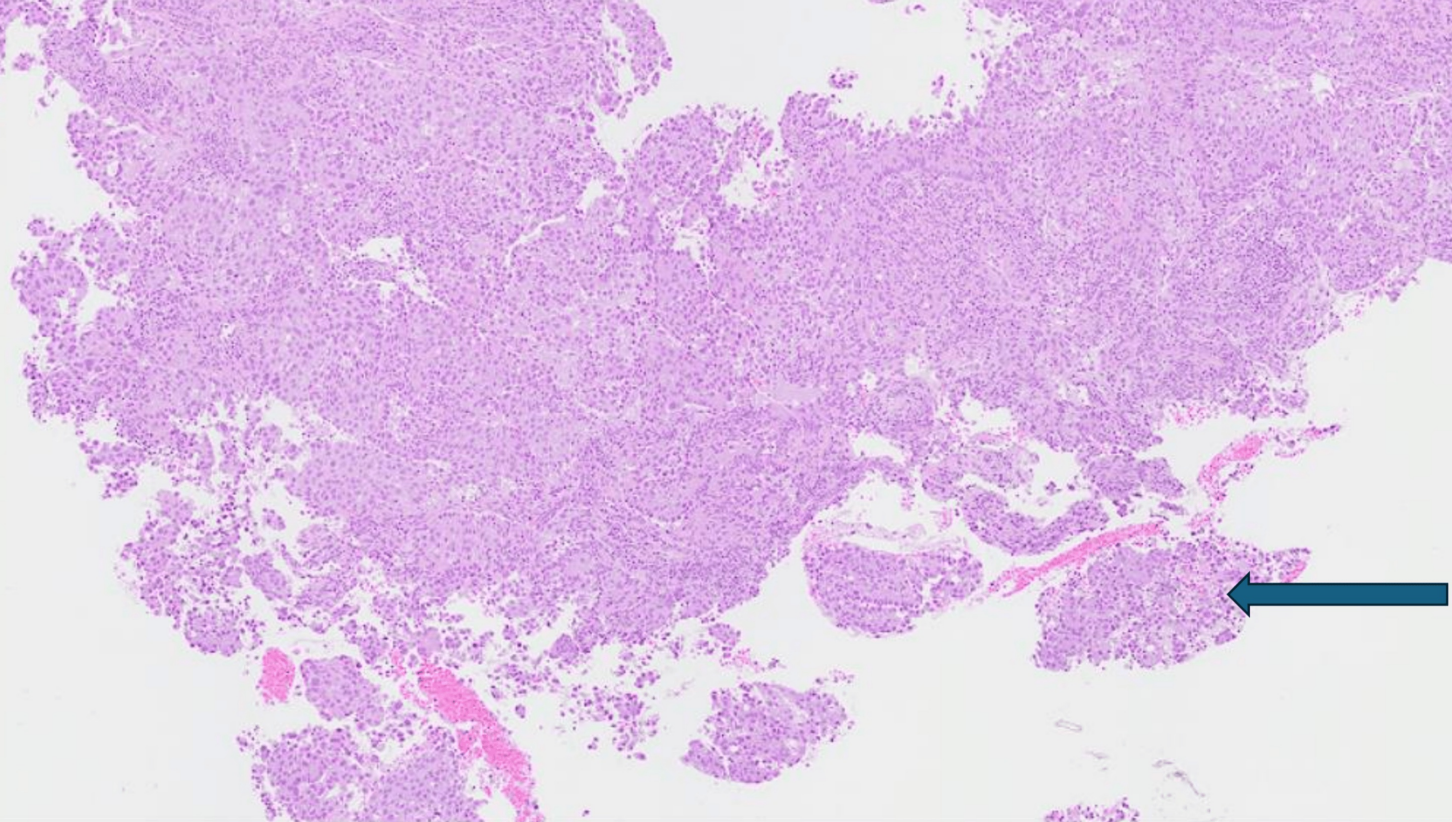
Low-power view of cervical tumor tissue. Hematoxylin and eosin (H&E) stain, low magnification. The image shows an infiltrative cervical tumor with complex glandular architecture involving the cervical stroma (arrow). Tumor nests are irregular and dispersed throughout the stroma, consistent with an invasive pattern of adenocarcinoma.

**Figure 5:**
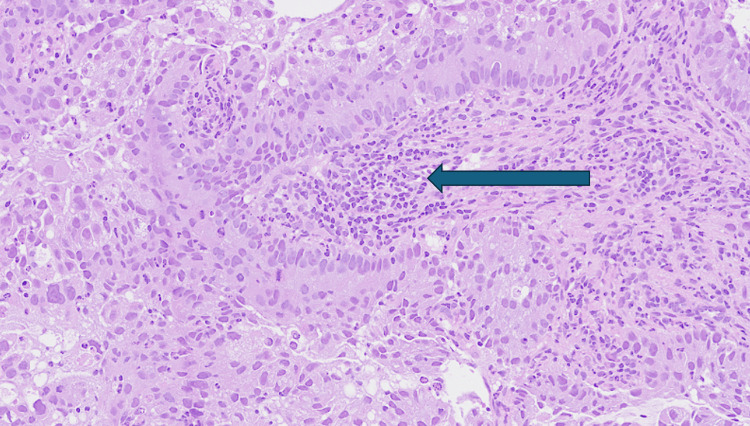
High-power view of cervical adenocarcinoma. Histopathology demonstrates irregular neoplastic glands with nuclear stratification, crowding, and atypia (arrow). These features are consistent with HPV-associated cervical adenocarcinoma. The infiltrative glandular architecture and marked nuclear abnormalities support a diagnosis of Silva pattern C, which correlates with an aggressive clinical course. HPV, human papillomavirus

Immunohistochemical staining showed strong positivity for carcinoembryonic antigen (CEA). Markers for p63, SMA, and desmin were negative, effectively excluding squamous and mesenchymal differentiation. These findings supported a diagnosis of HPV-associated cervical adenocarcinoma, classified as Silva Pattern C, characterized by destructive stromal invasion. Although no lymphovascular invasion (LVI) was detected in the biopsy sample, Silva Pattern C is associated with a higher risk of LVI, recurrence, and poorer prognosis. Key histological features are summarized in Table 4, with representative high-power images presented in Figures 4-5.

**Table 4:** Histopathology and immunohistochemistry findings Histology and immunohistochemistry confirmed HPV-associated cervical adenocarcinoma with Silva pattern C, without lymphovascular invasion. CEA, carcinoembryonic antigen; SMA, smooth muscle actin; HPV, human papillomavirus

Feature	Findings
Tumor morphology	Sheets of round to polygonal cells with eosinophilic to focally clear cytoplasm; moderately pleomorphic nuclei; focal nuclear palisading; atypical mitoses; necrosis; desmoplastic stromal reaction
Architectural pattern	No definite gland formation or papillary structures
Silva classification	Pattern C (destructive stromal invasion)
Lymphovascular invasion	Not identified
Immunohistochemistry	CEA: strongly positive; p63: negative; SMA: negative; Desmin: negative
Final diagnosis	HPV-associated adenocarcinoma of the cervix, Silva pattern C

The case was reviewed and discussed in a multidisciplinary team (MDT) meeting comprising gynecologic oncology, radiology, pathology, and oncology specialists. Given the FIGO stage IIIC2 disease with para-aortic lymph node involvement, the consensus was to proceed with definitive chemoradiotherapy. The patient was subsequently referred to the oncology center for concurrent chemoradiation and ongoing follow-up.

This case underscores the diagnostic challenges of endocervical adenocarcinoma, which often evades early detection by conventional cytology screening methods. Its tendency to present at an advanced stage contributes to a poorer prognosis compared to squamous cell carcinoma, reinforcing the need for improved screening strategies and public health awareness.

## Discussion

Cervical cancer remains a significant global health burden, particularly in low- and middle-income countries, where access to screening and HPV vaccination remains limited [1,2]. This case is a clear example of the consequences of missed screening opportunities, and low screening uptake remains a challenge, even in high-income countries like the United Kingdom. The patient, a 39-year-old British woman, had never attended cervical screening and presented with advanced-stage HPV-associated cervical adenocarcinoma. Despite the UK’s robust cervical cancer prevention infrastructure, including free access to screening and HPV vaccination, uptake remains suboptimal among certain populations. Factors such as embarrassment, fear, and logistical challenges can result in screening default, even within well-resourced healthcare systems [4].

To address these gaps, recent studies have explored the use of HPV self-sampling as a strategy to improve participation. The HOME randomized clinical trial demonstrated that mailing HPV self-collection kits significantly increased cervical cancer screening uptake compared with usual care reminders alone, confirming that structured strategies incorporating HPV self-sampling improve screening participation across diverse settings [5]. Further supporting this, the My Body, My Test-3 trial showed that mailing HPV self-collection kits led to higher engagement in cervical screening programs compared to standard outreach [6]. These findings emphasize the importance of adopting patient-centered, accessible interventions in national screening programs. Notably, immigrant and underserved populations may face additional cultural or systemic barriers. A recent systematic review and meta-analysis found that multicomponent interventions as combining education, community engagement, and self-sampling-significantly improved screening uptake among immigrants [7].

While squamous cell carcinoma is the most prevalent histologic subtype, adenocarcinoma now accounts for 10%-25% of cervical cancer cases and is increasing in incidence, particularly among younger women [12]. Persistent infection with high-risk HPV strains, especially HPV type 18, plays a key role in the pathogenesis of cervical adenocarcinoma [8,15].

Unlike squamous carcinoma, adenocarcinoma tends to originate higher in the endocervical canal, making it less likely to be detected through conventional cytology-based screening [8]. This case is a clear example of the consequences of missed screening opportunities. Our patient had never participated in cervical screening and presented with severe anemia secondary to abnormal uterine bleeding. Imaging revealed FIGO stage IIIC2 cervical adenocarcinoma, already involving para-aortic lymph nodes, highlighting the typically late diagnosis associated with this subtype.

Recent studies have emphasised key biological and clinical differences between HPV-related and HPV-independent adenocarcinomas, with HPV-independent types (often gastric-type) associated with worse prognosis and resistance to treatment [16,17]. Histological subtyping and Silva pattern classification have emerged as vital prognostic tools. In our case, the biopsy revealed Silva pattern C adenocarcinoma, which is associated with destructive stromal invasion, lymphovascular space invasion, and higher recurrence risk [16].

MRI played a critical role in staging, offering superior accuracy over CT in assessing tumor size, parametrial involvement, and nodal disease. Although CT scans can detect bulky lesions and nodal metastases, MRI remains the gold standard for local staging [12]. This patient’s MRI confirmed locally advanced disease, justifying treatment with extended-field chemoradiotherapy in accordance with FIGO 2018/2021 guidelines [12,14].

Histopathologically, the tumor exhibited Silva pattern C, characterized by diffuse destructive stromal invasion and lymphovascular space involvement. This pattern is associated with a higher risk of lymph node metastases and poorer prognosis, necessitating definitive treatment with extended-field chemoradiotherapy [16,17]. The classification of cervical adenocarcinoma into HPV-related and HPV-independent subtypes further refines prognosis and therapeutic decision-making, with HPV-independent types, such as the gastric subtype, carrying significantly worse outcomes and reduced responsiveness to radiation therapy [16].

In light of these findings, this case underscores the critical need for adaptable, inclusive strategies to enhance cervical cancer screening participation even in high-income countries. Broader implementation of self-sampling and culturally sensitive outreach programs could help reduce diagnostic delays and improve outcomes. As adenocarcinomas often present at more advanced stages and may be relatively resistant to radiation, early detection through HPV-based screening, greater awareness of non-cytological presentations, and an MDT approach remain essential to ensure timely diagnosis, accurate staging, and adherence to evidence-based treatment pathways.

**Figure 6:**
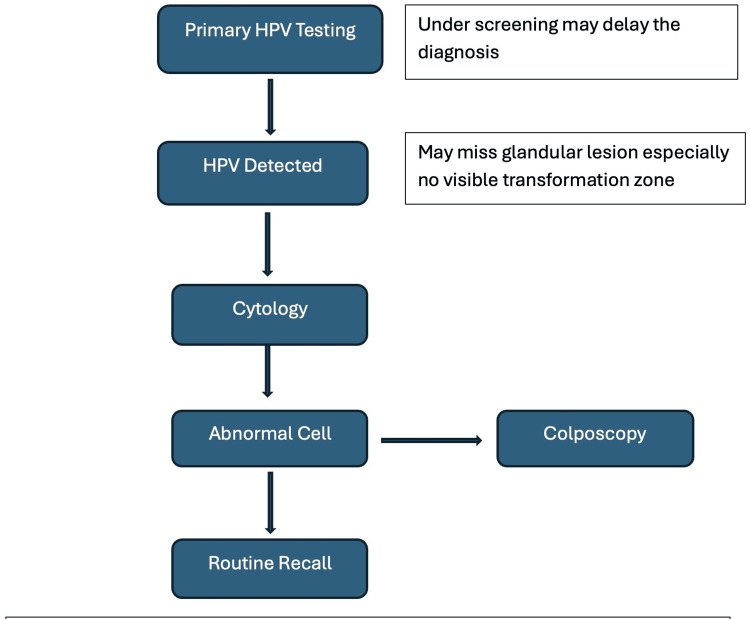
Conventional screening algorithm for cervical adenocarcinoma. This pathway relies on HPV testing followed by cytology and colposcopy, but may miss endocervical lesions, particularly when the transformation zone is not visible. Image credit: Created by the authors using Microsoft Word, based on UK cervical screening guidelines. ECC, endocervical curettage; HPV, human papillomavirus

To facilitate comparison between recommended and real-world diagnostic approaches, Figure 6 outlines the conventional cervical screening algorithm used in many high-income countries, including the United Kingdom [3]. This pathway, while effective for squamous lesions, may miss endocervical glandular lesions, especially when the transformation zone is not fully visualized or when cytology fails to detect abnormalities. 

**Figure 7:**
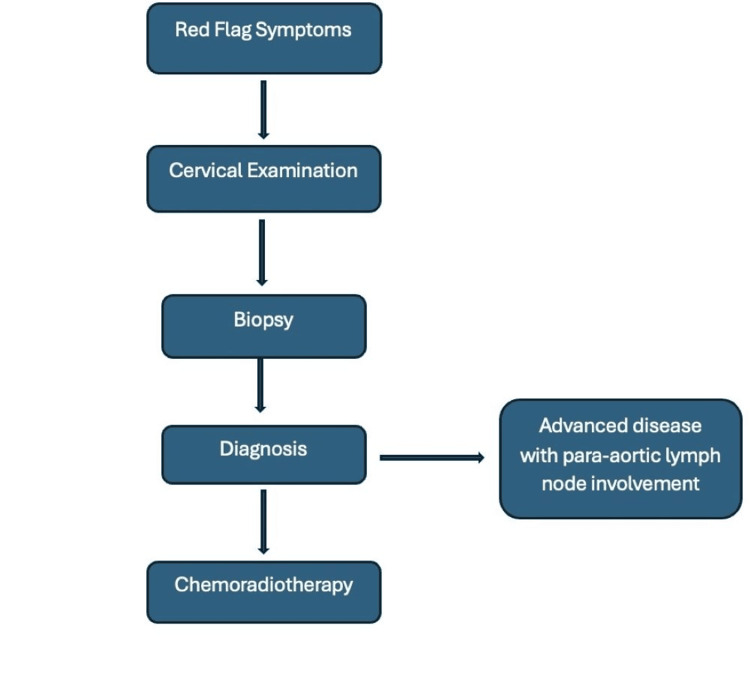
Diagnostic and staging approach for adenocarcinoma of the cervix. The patient presented symptomatically and underwent biopsy, imaging, and MDT review, bypassing the screening system due to non-attendance. Image credit: Created by the authors using Microsoft Word, based on the clinical management approach. MDT, multidisciplinary team

Figure 7 illustrates the diagnostic and staging steps undertaken in the current case, which was detected following symptomatic presentation. Presenting both flowcharts side by side allows clinicians to appreciate how defaulted screening and atypical symptomatology can alter the diagnostic trajectory and underscores the importance of maintaining a high index of suspicion for cervical adenocarcinoma.

## Conclusions

This case highlights the consequences of defaulted cervical screening, even in high-income countries with established prevention programs, particularly for glandular lesions like adenocarcinoma that often escape early cytological detection. MRI was indispensable for precise local and nodal staging, while histopathology confirmed Silva pattern C adenocarcinoma, an aggressive subtype associated with poorer prognosis. MDT input facilitated timely, guideline-based referral for definitive chemoradiotherapy.

These challenges reinforce the need for more accessible and inclusive screening approaches to prevent such late presentations. Strategies such as HPV self-sampling, community outreach, and culturally tailored education offer promising solutions to increase participation and reduce screening disparities. Ultimately, early recognition of red-flag symptoms combined with improved public awareness is essential to halt the silent progression of cervical adenocarcinoma.
